# Metagenomic Insights Into the Structure and Function of Intestinal Microbiota of the Hadal Amphipods

**DOI:** 10.3389/fmicb.2021.668989

**Published:** 2021-06-07

**Authors:** Jiulin Chan, Daoqiang Geng, Binbin Pan, Qiming Zhang, Qianghua Xu

**Affiliations:** ^1^Key Laboratory of Sustainable Exploitation of Oceanic Fisheries Resources, Ministry of Education, College of Marine Sciences, Shanghai Ocean University, Shanghai, China; ^2^Shanghai Engineering Research Center of Hadal Science and Technology, College of Marine Sciences, Shanghai Ocean University, Shanghai, China; ^3^Shanghai Rainbowfish Ocean Technology Co., Ltd, Shanghai, China; ^4^National Distant-water Fisheries Engineering Research Center, Shanghai Ocean University, Shanghai, China

**Keywords:** hadal trench, amphipod, gut microbiota, metagenomics, adaptation

## Abstract

Hadal trenches are the deepest known areas of the ocean. Amphipods are considered to be the dominant scavengers in the hadal food webs. The studies on the structure and function of the hadal intestinal microbiotas are largely lacking. Here, the intestinal microbiotas of three hadal amphipods, *Hirondellea gigas*, *Scopelocheirus schellenbergi*, and *Alicella gigantea*, from Mariana Trench, Marceau Trench, and New Britain Trench, respectively, were investigated. The taxonomic analysis identified 358 microbial genera commonly shared within the three amphipods. Different amphipod species possessed their own characteristic dominant microbial component, *Psychromonas* in *H. gigas* and *Candidatus Hepatoplasma* in *A. gigantea* and *S. schellenbergi*. Functional composition analysis showed that “Carbohydrate Metabolism,” “Lipid Metabolism,” “Cell Motility,” “Replication and Repair,” and “Membrane Transport” were among the most represented Gene Ontology (GO) Categories in the gut microbiotas. To test the possible functions of “Bacterial Chemotaxis” within the “Cell Motility” category, the methyl-accepting chemotaxis protein (*MCP*) gene involved in the “Bacterial Chemotaxis” pathway was obtained and used for swarming motility assays. Results showed that bacteria transformed with the gut bacterial *MCP* gene showed significantly faster growths compared with the control group, suggesting MCP promoted the bacterial swimming capability and nutrient utilization ability. This result suggested that hadal gut microbes could promote their survival in poor nutrient conditions by enhancing chemotaxis and motility. In addition, large quantities of probiotic genera were detected in the hadal amphipod gut microbiotas, which indicated that those probiotics would be possible contributors for promoting the host’s growth and development, which could facilitate adaptation of hadal amphipods to the extreme environment.

## Introduction

The hadal zone is the deepest area extending from 6,000 to 11,000 m depth from the ocean surface. It breaks the continuity of the abyssal plains and forms long but narrow topographic V-shaped ultra-deep habitats that occupy more than 45% of the total vertical depth of the marine environment (Lauro and Bartlett, 2008; Jamieson et al., 2009; Jamieson, 2015). In general, the hadal zone is an extreme environment characterized by low temperature, poor food resources, and high hydrostatic pressure (Bartlett, 1992). On the basis of extensive deep-sea sampling records, a wide range of organisms was found populating this extreme environment (Danovaro et al., 2002; Nunoura et al., 2015; Tarn et al., 2016). The organisms endemic to hadal zones attract wide interest in the specific types of physiological and biochemical adaptations necessary for growth and survival in the hadal habitats (Wolff, 1970; Marquis, 1982; Jannasch and Taylor, 1984; Wang et al., 2007). However, the survival mechanisms of the hadal organisms are so far poorly understood.

With increasing sampling efforts over extensive bathymetric ranges, amphipods (Arthropoda: Crustacea: Amphipoda) are being found to have cosmopolitan distributions and are considered to be the dominant scavengers in the hadal food webs (Eustace et al., 2013; Lacey et al., 2016; Linley et al., 2017). The large numbers and wide distributions of amphipods reflect their success in adapting to extreme hadal environments. Hadal amphipods have therefore become the main subjects of hadal environmental adaptation studies in recent years (Ritchie et al., 2015; Eustace et al., 2016; Ritchie et al., 2016). The hadal amphipods have proven to have special mandibles and capacious guts, which are considered to enable them to improve the utilization of sporadic food falls and to be adapted for bursts of feeding activity followed by prolonged periods of digestion and fasting (Ingram and Hessler, 1983; Saint-Marie, 1992). This lifestyle is thought to serve as an energy storage mechanism in hadal amphipods and has been speculated to resist long periods of food deprivation in the hadal environment (Buhring and Christiansen, 2001).

The gut microbiota has been shown to be involved with a number of aspects of host physiology, such as upgrading the nutrient status, developing of host immune system, and enhancing the ability of environmental adaptation in various organisms (Gil et al., 2004; Dugas et al., 2016; Zhang et al., 2016). Specifically, the presence of various bacteria in the guts of hadal invertebrates was proven to enhance the digestion of refractory organic compounds, which presumably provided another source of energy for amphipod individuals (Fang et al., 2000, 2003). Microorganisms, besides providing continuous energy for the hadal host, can also contribute specific essential nutrients, such as large quantities of essential fatty acids, essential amino acids, and important secondary metabolites (Phillips, 1984; Monica et al., 2008; Shulse and Allen, 2011). Psychrophilic and piezophilic bacteria have been reported to be isolated from the guts of deep-sea invertebrates in recent researches (Forget and Juniper, 2013; Goffredi et al., 2014). For example, *Hepatoplasma* spp., as a vast genus of deep-sea bacteria, has been proven to improve host survival under low nutrient conditions in hadal zones (Fraune and Zimmer, 2008). In studies on biological mechanisms of adaptation to extreme conditions, some microorganisms, especially deep-sea bacteria such as *Shewanella*, *Photobacterium*, *Colwellia*, *Vibrio*, and *Psychromonas*, have been shown to produce polyunsaturated fatty acids, which might play a vital role in low-temperature and high-pressure environments by resulting increase in membrane fluidity (DeLong and Yayanos, 1986; Fang et al., 2004; Nogi et al., 2007).

Metagenomics is the study of the collective genomes of microorganisms present in an environment. Under recent advances in high throughput sequencing, metagenomic analysis has become a more feasible technology to broaden and advance our ability to understand complex microbial communities and the association between microbial communities and specific environmental factors (Gurgui and Piel, 2010; Burke et al., 2011; Nemergut et al., 2013; Louca et al., 2016). Not much is known about the ecology and evolution of these hadal bacteria, and information about the structure and function of the hadal macro-organisms’ intestinal microbiotas are lacking. The main objectives of this study were to explore the total bacterial community and functional diversity associated with the guts of three different hadal amphipods by using the Illumina NovaSeq platform, aiming to provide supporting evidence of adaptive functions of the gut microbiota in hadal amphipods.

## Materials and Methods

### Sample Collection

**FIGURE 1:**
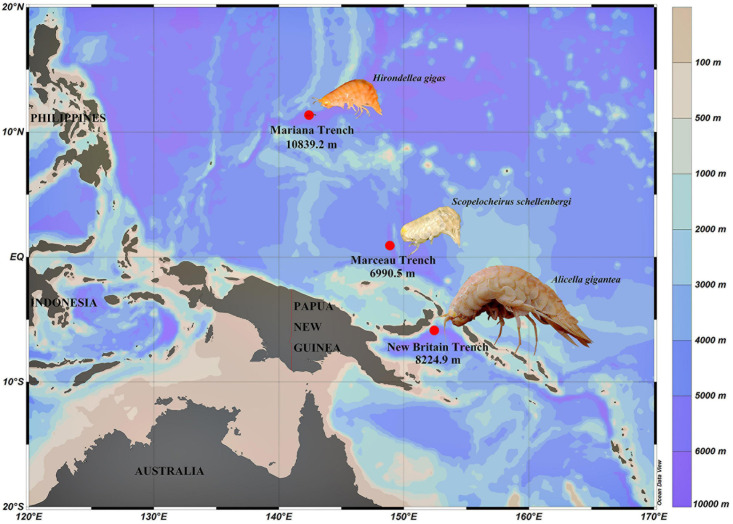
Sampling sites of three amphipods used in this study. *H. gigas* individuals were from the Mariana Trench (10,839.2 m). *S. schellenbergi* individuals were from Marceau Trench (6,990.5 m). *A. gigantea* individuals were from New Britain Trench (8,224.9 m). Color bar indicates different water depths.

Three species of amphipods, *Hirondellea gigas*,*Scopelocheirus schellenbergi*, and *Alicella gigantea*, were collected from the Mariana Trench, Marceau Trench, and New Britain Trench, respectively (Figure 1). The autonomous deep-ocean lander vehicle was equipped with two cage traps baited with a suitable amount of mackerels. The lander vehicle was launched from the “Zhang Jian” research vessel and deployed to the seafloor for up to 10 h. The detailed information about the lander vehicle and sampling was described in Chan et al. (2020). Upon recovery of the lander, amphipods were preserved immediately at –80°C until processed for analysis.

### DNA Extraction, Library Construction, and Metagenomics Sequencing

DNA for metagenomics was extracted from nine amphipods intestinal mucous samples (three individuals of each amphipod species) by using the FastDNA^®^ SPIN Kit for Feces (MP Biomedicals, Santa Ana, CA, United States) according to manufacturer’s protocols. Concentration and purity were quantified with TBS-380 and NanoDrop2000, respectively. DNA quality was detected by a 1% agarose gel electrophoresis system to ensure no degradation of genome DNA.

DNA extracts were fragmented to an average size of approximately 300 bp using Covaris M220 (Gene Company Limited, China) for paired-end library construction. A paired-end library was constructed using NEXTFLEX^®^ Rapid DNA-Seq (Bioo Scientific, Austin, TX, United States). Adapters containing the full complement of sequencing primer hybridization sites were ligated to the blunt-end of fragments. Paired-end sequencing was performed on Illumina NovaSeq (Illumina Inc., San Diego, CA, United States) at Majorbio Bio-Pharm Technology Co., Ltd., (Shanghai, China) using NovaSeq Reagent Kits according to the manufacturer’s instructions^1^. Sequence data associated with this project have been deposited in the National Center for Biotechnology Information (NCBI) Sequence Read Archive (SRA) database (BioProject accession number: PRJNA648173; SRA accession number: SRR12315170-SRR12315178).

### Sequence Quality Control and Genome Assembly

Adapter sequences were stripped from the 3′ and 5′ end of paired-end Illumina reads using SeqPrep^2^. Low-quality reads (reads with N bases, a minimum length threshold of 50 bp, and a minimum quality threshold of 20) were removed by Sickle^3^. Reads were aligned to an available amphipod (*Hyalella Azteca*) genome^4^ by BWA (Li and Durbin, 2009) (^5^, version 0.7.9a), and any hit associated with the reads and their mated reads were removed.

Succinct de bruijn-graph-based assembler MEGAHIT (Li et al., 2015) (^6^, Version 1.1.2) (parameters: kmer_min = 47, kmer_max = 97, step = 10) was used to assemble short reads at different sequencing depths. K-mers, using iterations of reads 47 to 97 bp in length, were tested for each sample. Scaffolds with a length of more than 500 bp were retained for statistical tests. We evaluated the quality and quantity of scaffolds generated by each assembly and finally chose the best K-mer that yielded the minimum scaffold number and the maximum value of N50 and N90. Then, scaffolds with a length of more than 500 bp were extracted and broken into contigs without gaps. Contigs were used for further gene prediction and annotation.

### Gene Prediction, Taxonomy, and Functional Annotation

Open reading frames (ORFs) from the assembled contigs were predicted using MetaGene (Noguchi et al., 2006). Genes with nucleic acid length ≥ 100 bp were selected and translated into an amino acid sequence to obtain the gene prediction results of each sample by using the NCBI translation table^7^.

All predicted genes with a 95% sequence identity (90% coverage) were clustered using CD-HIT (Fu et al., 2012) (^8^, version 4.6.1). The longest sequences from each cluster were selected as representative sequences to construct a non-redundant gene catalog. Reads after quality control (length threshold is greater than 50 bp and quality threshold is greater than 20) were mapped to the representative sequences with 95% identity using SOAPaligner (Li et al., 2008) (version 2.21), and gene abundance for each sample was evaluated. Read counts were normalized by the method of reads per kilobase per million mapped reads.

Representative sequences of non-redundant gene catalog were aligned to NCBI NR database with an e-value cutoff of 1e-5 using BlastP (^9^, version 2.2.28+) (Altschul et al., 1997) for taxonomic annotations. The Kyoto Encyclopedia of Genes and Genomes (KEGG) and probiotics annotation were conducted using BlastP against the KEGG database (Xie et al., 2011) (^10^, version 96.0) and Probio database with an e-value cutoff of 1e-5, respectively.

### Phylogenetic and Domain Composition Analysis of Methyl-Accepting Chemotaxis Protein*s*

Methyl-accepting chemotaxis protein (*MCP*) complete sequences of the microorganisms were obtained in NCBI databases by blasting, and 16 related sequences were downloaded. Sequence alignments were generated using Clustal W and then imported into MEGA X software for phylogenetic analysis using the maximum likelihood method with a bootstrap replication number of 1,000. Protein sequences of these *MCPs* were downloaded from NCBI and compared using the Clustal W program. Simple Modular Architecture Research Tool^11^ was used to predict protein domains.

### Plasmid Construction, Protein Expression, and Swarming Motility Assays

The *MCP* with perfect ORF was obtained from the predicted ORFs of our metagenomic dataset and was subsequently used in swarming motility assays. The sequence of the *MCP* gene has been deposited into the NCBI with GenBank accession number: MT792534. The *MCP* recombinant expression plasmids were constructed using pET-32a vector (TIANGEN, China) with restriction endonucleases EcoR I and Not I (TAKARA, Japan) and then checked by DNA sequencing and restriction enzyme digestion. For the expression of the *MCP* proteins, the recombinant expression plasmids were transformed into *Escherichia coli* BL21 (DE3) competent cells (TIANGEN, China). Positive transformants were picked from single colonies on Luria–Bertani (LB) plates and grown overnight at 37°C in 15 ml of LB medium with 30 μg/ml kanamycin. The cultured cells were then inoculated to 20-ml fresh LB medium (1:50 dilution) containing 50 μg/ml of kanamycin at 37°C until the optical density of the sample measured at a wavelength of 600 nm values reached 0.6, followed by the addition of isopropyl-β-D-1-thiogalactopyranoside (IPTG) (TIANGEN, China) to the final concentration of 1 mM to induce the expression of MCP proteins. Recombinant *MCP* expressions were tested by western blot analysis using anti-6 × His tag (1:3,000, BBI Biotech, Germany).

Swarming motility assays were carried out with soft agar plates (Sarand et al., 2008) with soft agar plates containing 0.5% agarose, 1% sodium chloride, 50 μg/ml kanamycin, and 1-mM IPTG in gradient nutrient mediums. Mediums with five different nutrient concentrations were used in current assays, which were 100% (contains 1% peptone, 0.5% yeast extract), 80% (contains 0.8% peptone, 0.4% yeast extract), 60% (contains 0.6% peptone, 0.3% yeast extract), 40% (contains 0.4% peptone, 0.2% yeast extract), and 20% (contains 0.2% peptone, 0.1% yeast extract). Bacterial cells induced by IPTG from LB cultures were inoculated into the solidified agar and incubated at 26°C for 20 h, followed by image collection.

## Results

### Community Structures of the Hadal Amphipod Gut Microbiota

**FIGURE 2:**
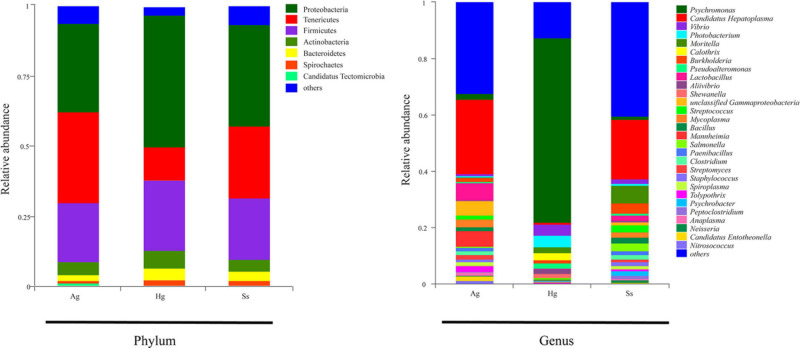
Relative abundance of bacterial communities in intestine samples of hadal amphipods. Community compositions are displayed at phylum level and genus level. Genera totaling < 1% of samples were assigned as “Others.” Ag (*A. gigantea*), Hg (*H. gigas*), and Ss (*S. schellenbergi*) stand for different amphipod species.

**FIGURE 3:**
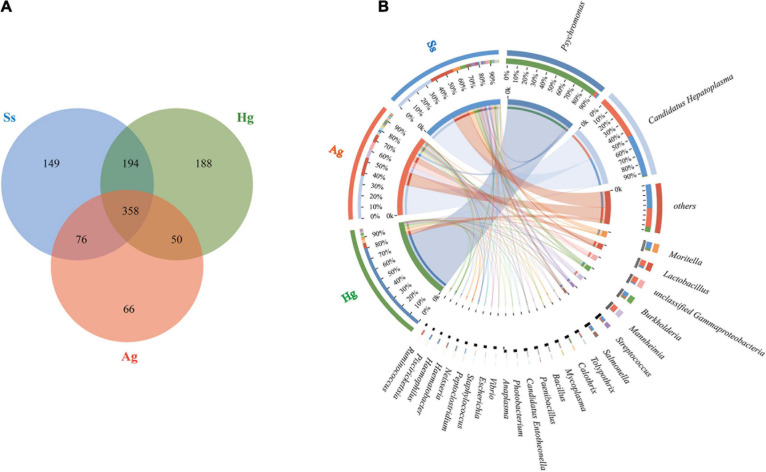
Microbial diversity in three hadal amphipod groups. **(A)** Venn diagram illustrating total, unique, and shared numbers of genera predicted from three datasets. **(B)** Circos diagram illustrating genus-level microbial composition in all samples.

Nine DNA samples extracted from the intestinal mucous of three species of amphipod *H. gigas, S. schellenbergi*, and *A. gigantea* were used to sequence the gut microbiota, generating metagenomic data sets totaling 78.23 Gb. Read numbers, quality filtering, and the assemblies of Illumina sequencing were summarized in Supplementary Tables 1, 2. The host (amphipod) derived reads have been removed before analysis, and the relevant information is shown in the optimized reads in Supplementary Table 1. *De novo* assembly of effective reads resulted in 32,648 non-redundant catalog genes, which were used for subsequent analysis. Taxonomic classification based on non-redundant catalog genes revealed a total eight phyla (with > 1% relative abundance of the total microorganisms) from the gut microbial communities. Taxonomic profiling indicated that the hadal amphipods’ gut microbiota was dominated by Proteobacteria, followed by Tenericutes and Firmicute*s* and, to a lesser extent, Actinobacteria, Bacteroidetes, Spirochaetes, and Candidatus Tectomicrobia (Figure 2). At the genus level, a total of 1,081 genera were identified, of which 358 were shared by the three amphipod species (Figure 3A). Overall, *Candidatus Hepatoplasma* was the most abundant genus in the gut microbiota of both the New Britain Trench *A. gigantea* and the Marceau Trench *S. schellenbergi* individuals, whereas *Psychromonas* was more abundant in the Mariana Trench *H. gigas* individuals (Figures 2, 3B).

### Functional Profiles of the Hadal Amphipod Gut Microbiomes

**FIGURE 4:**
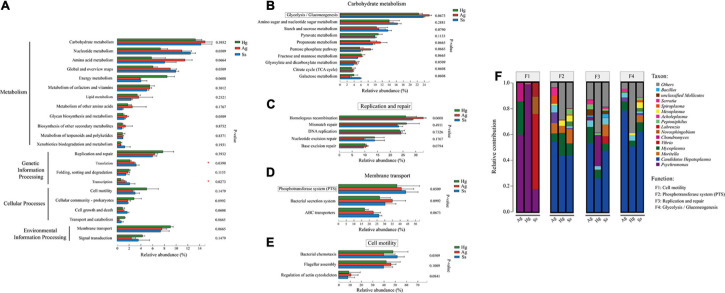
Functional composition and comparison of hadal amphipod gut microbiomes. **(A)** KEGG classification of total non-redundant protein-coding genes from all intestinal samples. “Metabolisms,” “Genetic Information Processing,” “Cellular Processes,” and “Environmental Information Processing” were most presented functions at KEGG Level 1 pathway (SEED1). Rightmost is *P*-value. **(B–E)** Most presented KEGG level 2 pathways (SEED2) among four SEED1 categories [shown in panel **(A)**], “Carbohydrate Metabolism,” “Replication and Repair,” “Membrane Transport,” and “Cell Motility,” respectively. Extended error bar plot showed differences of functional categories at KEGG level 2 between three hadal amphipod species. Different colors indicate different groups. Rightmost is *P*-value: *P < 0.05. Four KEGG pathways, “Glycolysis/Gluconeogenesis,” “Replication and Repair,” “Membrane Transport” (PTS), and “Cell Motility,” marked in red box, were selected out for Species Contribution Relationship Analysis. **(F)** Species and functional contribution analysis. Species contribution relationship of four KEGG functional categories at gene level.

The gene functions annotated by KEGG pathway hierarchical level 1 (SEED 1) that indicate “Metabolisms” were the predominant function in the total non-redundant protein-coding genes (SEED1 in Figure 4A), followed by “Genetic Information Processing,” “Cellular Processes,” and “Environmental Information Processing” (Figure 4A). “Carbohydrate Metabolism” (13.29, 14.92, and 14.22% of the total KO function in gut microbiota of the *H. gigas*, *A. gigantea*, and *S. schellenbergi*, respectively), “Replication and Repair” (8.13, 6.72, and 6.08% for *H. gigas*, *A. gigantea*, and *S. schellenbergi*, respectively), “Cell Motility” (5.08, 2.68, and 3.17% for *H. gigas*, *A. gigantea*, and *S. schellenbergi*, respectively), and “Membrane Transport” (9.42, 7.87, and 7.62% for *H. gigas*, *A. gigantea*, and *S. schellenbergi*, respectively) were detected as the most presented KEGG pathway hierarchical level 2 (SEED 2) types among the four SEED1 categories (Figure 4A).

The top 3–10 abundant KEGG pathway hierarchical level 3 (SEED3) for the four SEED2 categories (“Carbohydrate Metabolism,” “Replication and Repair,” “Cell Motility,” and “Membrane Transport”) were shown separately (Figures 4B–D), in which “Glycolysis/Gluconeogenesis” was detected as the most abundant category in “Carbohydrate Metabolism” (22.46, 25.40, and 23.83% of the total “Carbohydrate Metabolisms” functional activities in the gut microbiota of the *H. gigas*, *A. gigantea*, and *S. schellenbergi*, respectively, see Figure 4B). “Homologous Recombination” was the main pathway in “Replication and Repair,” among which 26.02, 34.98, and 28.31% of the total “Replication and Repair” functional activities were in the gut microbiota of the *H. gigas*, *A. gigantea*, and *S. schellenbergi*, respectively (Figure 4C). Moreover, the “Bacterial Chemotaxis” and “Phosphotransferase System” (PTS) were acted as the most presented categories of “Cell Motility” and “Membrane Transport,” respectively (Figures 4D,E). It should be noted that the genes involved in “Flagellar Assembly” were the second most abundant category in the “Cell Motility,” ranging from 39.97 to 46.78% of the total “Cell Motility” functional activities in the gut microbiota of three hadal amphipod samples (Figure 4E).

In addition, the categories of “Metabolic and Cellular Processes,” “Cell,” “Cell Part,” and “Membrane and Membrane Part” were also identified in the Gene Ontology (GO) classification based on the read sets of three hadal amphipod species (Supplementary Figures 1, 2).

### Functional Comparisons of the Hadal Amphipod Gut Microbiomes

To measure the difference between the microbiota functions of the three hadal amphipod groups, we analyzed their diverse functional compositions based on the Kruskal–Wallis and post-hoc tests. The results of a comparative analysis of the compositions and relative abundances of KEGG pathways in the three hadal amphipod gut microflora showed that except for “Translation” and “Transcription” (*P*-value > 0.05, Figure 4A), there were no significant functional differences detected in all the SEED2 functional compositions (Figure 4A). Similar results were also found in the SEED3 functional compositions of “Carbohydrate Metabolism,” “Replication and Repair,” “Cell Motility,” and “Membrane Transport” (*P*-value > 0.05; Figures 4B–E). Moreover, consistent with the results of KEGG functional composition comparisons, there were no differences detected in the GO functional composition among the three groups of gut microbes (*P*-value > 0.05; Supplementary Figure 2).

### Difference on the Species and Functional Contribution Relative Abundances Between Three Hadal Amphipod Species

Correlation analysis of the species and functional contribution relative abundances was performed in the samples from the three localities. In the species functional contribution analysis based on the four most presented KEGG terms, “Cell Motility,” “Phosphotransferase System (PTS),” “Replication and Repair,” and “Glycolysis/Gluconeogenesis,” we annotated the “Cell Motility” functional categories and found it was mainly contributed by *Psychromonas* in *H. gigas* and *A. gigantea* groups, whereas *Moritella* and *Vibrio* were detected to be the major contributors in the *S. schellenbergi* group (Figure 4F). In comparison, the main KEGG functional pathways of “Phosphotransferase System (PTS),” “Replication and Repair,” and “Glycolysis/Gluconeogenesis” were detected to be mainly contributed by *Candidatus Hepatoplasma*, followed by *Mycoplasma* in all the three hadal amphipod groups (Figure 4F). These different functional contributions suggested that the diverse composition of bacteria species possibly realized the hadal environmental adaptability of gut microbiomes from different hadal amphipods.

### Cell Motility and Functional Validation in the Gut Microbiota of Hadal Amphipods

**FIGURE 5:**
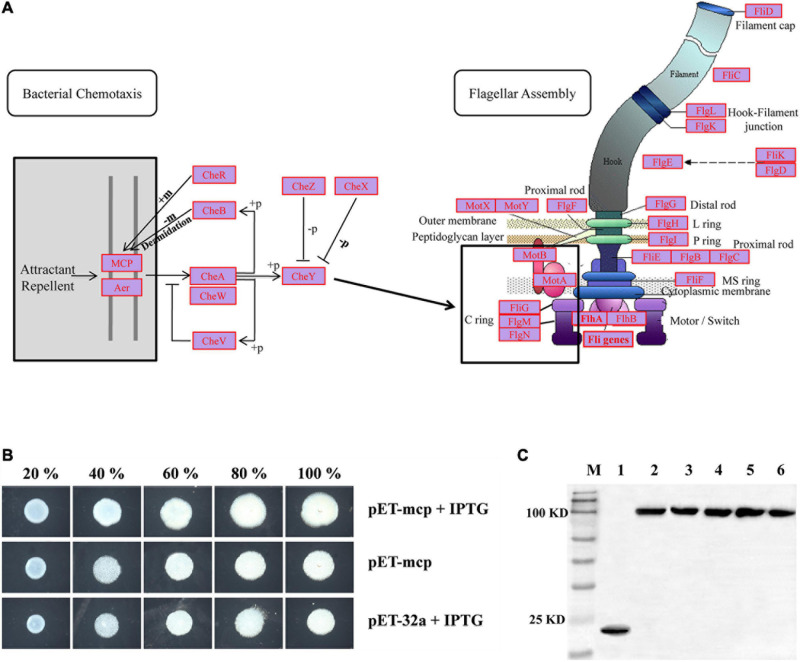
**(A)** Most presented genes involved in “Bacterial Chemotaxis” and “Flagellar Assembly” KEGG pathways. Most presented genes are indicated by red legend within complete pathways of “Bacterial Chemotaxis” (ko02030) and “Flagellar Assembly” (ko02040). Expression profiles of genes involved in “Bacterial Chemotaxis” and “Flagellar Assembly” KEGG pathways were shown on Supplementary Table 3. **(B)** Swarming motility of control-type and MCP expressed strains were examined on swarming permissive agar by spotting normalized cultures of indicated strains and photographed at 20 h. 20, 40, 60, 80, and 100% indicated five different nutrient concentrations used in assays. **(C)** Western blotting detects expression of MCP proteins. M: Marker; 1: Control strain; 2–6: MCP expressed strains in 20, 40, 60, 80, and 100% nutrient concentrations, respectively.

To gain more insight into possible functions of the “Cell Motility” category, the KEGG database was used for read-based alignments. For the hadal amphipod samples, a large number of genes related to “Cell Motility” were associated with KEGG level 3 pathways and specifically the “Bacterial Chemotaxis” and “Flagellar Assembly” (Figure 5A). Within the “Bacterial Chemotaxis” pathway, a macromolecular complex known as chemosensory arrays and assembled from the *MCP*, redox receptor Aer, histidine kinase CheA, and adaptor protein CheW was highly presented (Figure 5A marked in red, Supplementary Table 3). In addition, the gene encoding the CheY protein, which acts as a “Response Regulator” during signaling to the flagellum, was also detected to be highly presented (Figure 5A and Supplementary Table 3). Similarly, within the “Flagellar Assembly” pathway, almost all genes involved in this pathway were highly presented (see Supplementary Table 3), including *MotA*, *MotB*, *FliG*, *FliM*, and *FliN* genes (Figure 5A, marked in red, Supplementary Table 3), which are considered important factors of the non-rotating part of the flagellum-motor complex controlling flagellar rotation.

“Bacterial Chemotaxis” is the KEGG pathway associates with the microbes’ ability to control motility in responding to chemical environments, allowing bacteria to move toward a more favorable location and maintain a steady-state despite ambient conditions. To test the possible hadal adaptation functions of the “Bacterial Chemotaxis” pathway, the *MCP* gene within the “Bacterial Chemotaxis” pathway (Figure 5A) was obtained from the current metagenomic dataset. With respect to the phylogenetic tree, the sequence for *MCP* from the hadal amphipod was formed a sister clade approximate to the different microorganisms of the same genus in hadal amphipods (e.g., *Moritella*, *Shewanella*, *Vibrio*, *Photobacterium*, and *Pseudoalteromonas*). The hadal amphipod *MCP* was also separated from those associated with other species in the tree, such as the microorganism previously isolated from terrestrial and shallow water (Supplementary Figure 3). Moreover, based on protein domain prediction, the specific 3C8C| B domain was detected in the *MCP* from hadal amphipod (Supplementary Figure 3) and which was obviously different from other species in the tree. Finally, the *MCP* recombinant expression plasmids were constructed and subsequently introduced into *E. coli*. *E. coli* clones stably expressing the hadal bacterial *MCP* were found to be significantly enhanced in mobility activity and faster growth compared with the control group (Figures 5B,C). The result suggested a contribution by *MCP* to motility and growth of the gut microbiota in the hadal amphipods where nutrients are poor.

### Potential Strategies of Gut Microbiota Effects on Environmental Adaptation of Hadal Amphipods

**FIGURE 6:**
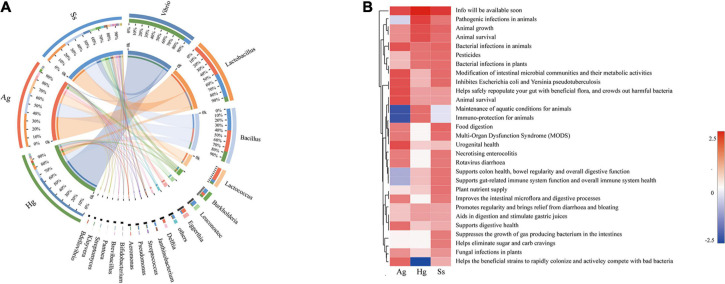
Composition and functional analysis of probiotics in intestine samples of hadal amphipods. **(A)** Relative abundance of probiotics at genus level of intestinal metagenomic samples from three hadal amphipods, Hg, Ss, and Ag. **(B)** Functional composition of probiotics.

The intestine is colonized by a wide variety of microbes that comprise the “gut microbiota,” a complex microbial community that has co-evolved with the host to form a mutually beneficial relationship *via* a dynamic balance of symbiosis and competition. Accumulating evidence indicates that the gut microbiota can participate in various life activities of the host, such as promoting the absorption of nutrients and energy storage, while also playing many complex roles in the immune function and environmental adaptability in the host (Belkaid and Hand, 2014; Ayres, 2016). Therefore, to explore the effects of gut microbiota on the hadal amphipods’ adaptations to the extreme environment, the Probio database was used for read-based alignments using the BLAST algorithm with a significant e-value threshold of 1e-5. According to the identifications of probiotics against predicted ORFs from our metagenome dataset, a series of probiotics genera such as *Lactobacillus*, *Bacillus*, *Lactococcus*, *Burkholderia*, *Leuconostoc*, *Bifidobacterium*, and *Bdellovibrio* were detected in large quantities in the hadal amphipod gut microbe samples (Figure 6A). Functional annotation showed that the main functions of these probiotic-related genes were associated with “Animal Survival,” “Animal Growth,” “Immune-protection,” and “Food Digestion” (Figure 6B), which indicated that those probiotics among the hadal amphipod gut microbiota could be contributors of host’s growth and development. In addition, the functional interaction analysis between probiotics and the host further showed the correlations between probiotic effects and categories of predicted gene functions in hadal amphipods, which indicated that they might play important roles in multiple functional pathways in the hadal amphipods, such as “Cell and Membrane Processes,” “Metabolism Processes,” “Cellular Processes,” “Localization and Binding,” and so on (Supplementary Figure 4).

## Discussion

The hadal zone is the deepest habitat on Earth, characterized by the near-freezing temperature, scarcity of food and nutrition, and elevated hydrostatic pressure (Lauro and Bartlett, 2008; Jamieson et al., 2010). These physically uniform environmental factors are believed to have contributed to the evolution and persistence of a variety of metazoan organisms (such as fish, polychaetes, and amphipods) and microorganisms, mainly consisting of psychrophilic and piezophilic bacteria, either free-living or host-associated (Sogin et al., 2006; Glud et al., 2013; Ichino et al., 2015; Lacey et al., 2016). In the previous studies of microbial communities of *trans-*trench sediments/water, the vertically distinct microbial community structures were detected between abyssal and hadal sediments/water and which indicated that hadal trenches contained unique microbial biodiversity (Peoples et al., 2018; Hiraoka et al., 2020). Moreover, the widespread distribution of amphipods in the hadal trenches reflects their successful adaptation to the hadal environments, which may also indicate that unique hadal environmental factors may be responsible for the evolution and presence of specific microbial species in the hadal amphipod gut. Although the well-recognized importance of microbes with regard to their biogeochemical roles in hadal zones, their adaptive strategies and the functional potential of animal association remain poorly studied. In the present study, we revealed the composition and function of gut microbiota of three amphipod species by a metagenomics approach. Our results unfolded the universal similarity between the three hadal amphipod populations, as determined by alpha and beta diversity analyses of the taxonomic compositions. Simultaneously, the analyses of the functional potentials revealed the preliminary environmental adaptation strategies for gut microbiota and the potential interactions between the host and gut microbiota in the process of adapting amphipods to hadal environments.

As we know, intestinal contents have been widely used for metagenomic analysis in several animal species (Suo et al., 2017; Zhao et al., 2018). In our study, based on the composition of the gut microbial communityfrom three hadal amphipod species, the common dominant taxa at phylum and genus levels were detected (Figures 2, 3), which suggested that the gut microbial compositions of the co-dwelling hadal amphipods were relatively constant inter-species. We noted that the dominant bacterial taxa at the genus level, such as *Candidatus Hepatoplasma* and *Burkholderia*, have been reported to be abundant in different populations of hadal amphipod species (Zhang et al., 2018, 2019; Cheng et al., 2019) and also been reported to be symbionts of bean bugs (Kim et al., 2015) and terrestrial isopods (Wang et al., 2004). *Candidatus Hepatoplasma*, identified as a *Mycoplasma*-like symbiont abundant in terrestrial isopods, showed a positive correlation between the host and survivorship on low-quality food (Sebastian and Martin, 2008). In the studies of Wang et al. (2004) and Fraune and Zimmer (2008), *Candidatus Hepatoplasma* was reported as a symbiont in the midgut glands of terrestrial isopods, with the results suggesting functions to be related to cellulase production for leaf litter degradation. This is a beneficial relationship for the host in low-nutrient conditions. Moreover, in the study of Sebastian and Martin (2008), most isopods that survived feeding on a cellulose-based low-quality diet for 90 days harbored *Candidatus Hepatoplasma* in their midgut glands, whereas those that died within 90 days mostly either harbored no or other bacterial symbionts. The presence of these microbial groups, therefore, suggests a close relationship between the gut microbiota and the hadal amphipod host. In addition, members of *Psychromonas* and *Pseudoalteromonas* genera were also detected to be highly represented in the gut microbiota of hadal amphipods in our study, especially *Psychromonas*, which has been shown to contain a variety of piezophilic and psychrophilic species that are widely distributed in marine environments, including hadal zones (Groudieva et al., 2003; Miyazaki et al., 2008). Members of these two genera are thought to share common features, such as synthesis of polyunsaturated fatty acids and/or expression of related genes to enhance membrane fluidity (Casanueva et al., 2010), which likely facilitates cold and pressure adaptation and survival of the hadal amphipods. However, differences in gut microbial composition were also detected among the three geographically isolated amphipod species (Figure 3), which could be explained by multiple factors such as the host genetic background, environmental location, and food support (Miyake et al., 2005; Wong and Rawls, 2012; Liu et al., 2016). For instance, *Candidatus Hepatoplasma* was detected as the dominant bacterium in the gut of the *S. schellenbergi* and *A. gigantea*, whereas relative absence in the *H. gigas.* Given that the New Britain Trench is closest to the coast followed by the Marceau Trench, and finally the Mariana Trench, the decaying plant material content in these trenches should show a decreasing trend, which may be the reason for the difference in the abundance of *Candidatus Hepatoplasma* in the gut of different amphipod species, and similar conclusions have been mentioned in previous studies (Zhang et al., 2019). Moreover, the difference in the abundance of *Psychromonas* of gut microbial composition was also detected among the three geographically isolated amphipod species, especially the almost absence of *Psychromonas* in the *S. schellenbergi* and *A. gigantea*, which suggested that their function is dispensable or their counterparts have emerged in the guts of these amphipods, as Zhang et al. (2019) mentioned in the previous study.

To further understand the environmental adaptations of gut microbes in the hadal trench, functional analyses were carried out. From the KEGG and GO annotation and classification analyses, it was obvious that non-differentiated functional components (except for “Translation” and “Transcription” in SEED2 functional composition) could be observed from the three geographically isolated amphipod samples in our study (Figure 4 and Supplementary Figure 2). In detail, the functional pathways of PTS, Glycolysis/Gluconeogenesis, Homologous Recombination, Bacterial Chemotaxis, and Flagellar Assembly were detected as the common functional types in all the amphipod gut samples, suggesting that the existence of a set of similar functional components in gut microbes of the hadal amphipod species (Figure 4 and Supplementary Figures 1, 2). However, the previous study on the *H. gigas* from Mariana Trench and Japan Trench have shown that different geographical locations lead to functional differentiation of their gut microbes (Zhang et al., 2019) and which was inconsistent with the results of the present study. This difference may result from a combination of selection factors, such as habitat environment, microbial interaction, and host genetic background, and the details remain to be analyzed.

The PTS is a complex group translocation system used by bacteria for transporting carbohydrates from cytoplasm to cytoplasm with the least energy-consuming and the most efficient way (Gosset, 2005), which can also play key roles in “Metabolism and Regulation” and provide the initial substrate for glycolysis, and by which bacteria can integrate their nutritional status with diverse environmental stimuli (Snyder et al., 2014; Hayes et al., 2017). Meanwhile, the function of the PTS is also inhibited by high concentrations of several metabolizable PTS sugars (Ye and Saier, 1996). Thus, the high presence of “PTS” and “Glycolysis/Gluconeogenesis” in the gut microbiota of the hadal amphipods pointed to the low carbon content in the three hadal trenches, consistent with a previous study by Zhang et al. (2019), which suggested that the gut microbes mainly generate large amounts of energy to maintain their normal life activities in a hadal environment with the extreme scarcity of food supply through enhancing the PTS and Glycolysis/Gluconeogenesis functional pathways.

Motility is another important adaptation for marine bacteria, in particular to avoiding grazing and for the continuous quest for nutrients, which is arguably the most pressure-sensitive cellular process in the surface-water prokaryotes (Grossart et al., 2001; Bartlett, 2002). In a hadal environment, the hunt for dissolved and particulate organic matter might explain a large number of *MCPs* present in the genomes of all the deep bath types (Azam and Long, 2001; Kiorboe and Jackson, 2001), including the hadal amphipods in the present study. *MCPs* are signal-transducing proteins that respond to gradients of chemicals in the environment, relaying a signal for directional swimming to the flagellar motor. These sensory systems must be able to detect miniscule changes in the surrounding chemistry to enable the cells to maximize their productivity and growth in environments of small amounts of spatially and temporally distributed food supplies (Wirsen and Molyneaux, 1999). Therefore, to clarify the role of chemotaxis in bacteria motility, the motility of the stably *MCP* recombinant expression clones was assessed with soft agar plate motility assays (Figures 5B,C). Larger areas of plaque were observed in *MCP* transfected cells in our study (Figures 5B,C), which provided evidence that *MCP* promotes the swimming capability and their nutrients utilization efficiency of the *E. coli* and also proved that hadal amphipods gut microbes could promote their survival in the nutrient-poor conditions of hadal trenches by enhancing chemotaxis and motility. In addition, the specific 3C8C| B domain was only detected in the hadal amphipod *MCP* in our study (Supplementary Figure 3) and which belongs to the periplasmic sensor-like domain superfamily and is a compound structure of the double cache domain that have been predicted to have a role in small-molecule recognition in a wide range of proteins (Anantharaman et al., 2001). Although the functionality of 3C8C| B is as yet unknown, it probably makes the *MCP* gene specialized to the hadal microbes, however, which needs to be verified by substantive validation in the further functional experiments.

Intestinal microbiomes are complex ecosystems, which act as the “extra organ” playing an important role in maintaining the host’s health (Heintz-Buschart and Wilmes, 2018). Numerous recent studies have shown that several intestinal functions are achieved through bacterial metabolism, which may benefit the host by improving pathogen defense, nutrient absorption, homeostasis maintenance, and immune response (Hooper and Macpherson, 2010; Iwase et al., 2010; Ridaura et al., 2013). Probiotics are an important part of intestinal microbiomes and are defined as live microorganisms that confer a benefit on the host and which could contribute to inhibit pathogenic microorganisms, increase the immune response, enzymatic digestion, and promote growth factors of organisms (Krummenauer et al., 2014). Our study revealed that *Lactobacillus*, *Bacillus*, *Lactococcus*, *Burkholderia*, and *Leuconostoc* were the predominant probiotic genera in the intestine of the hadal amphipods (Figure 6A), in which the genera *Lactobacillus*, *Lactococcus*, and *Leuconostoc* are included being representatives in the group of lactic acid bacteria. As has been described before, lactic acid bacteria are considered to be very important, as they could produce several antimicrobial substances (lactic acid, H_2_O_2_, bacteriocins, etc.), digestive enzymes (amylase, lipase, protease, etc.), and coenzymes (folate and cobalamin) (Carina et al., 2011; LeBlanc et al., 2011). Similarly, *Bacillus* and *Burkholderia* have also been shown to have the ability to produce a variety of digestive enzymes (amylase, lipase, protease, etc.) and antimicrobial substances (siderophores, pyrrolnitrin, monoterpenoid alkaloids, etc.) (Aly et al., 2008; Korkea-Aho et al., 2012). In addition, the genus *Vibrio* was the most dominant probiotic detected in this study. Although some *Vibrio* species, such as *V. anguillarum*, *V. coralliilyticus*, and *V. shiloi*, are considered as the pathogens of aquatic organisms (Austin and Zhang, 2006; Frans et al., 2011), most species from this genus are benign, such as *V. alginolyticus, V. campbellii*, and *V. fluvialis* detected in the present study, which has been reported to increase the non-specific immune level of the host and enhance resistance to disease (Thompson and Polz, 2006; Thompson and Swings, 2006). Moreover, based on the functional analysis of probiotics (Figure 6B) and the functional interaction analysis between probiotics and the host (Supplementary Figure 4), we also preliminarily confirm the interactions between intestinal microorganisms and the hadal amphipods. Therefore, the predominance of these probiotics in the hadal amphipod gut would be considered as possible contributors for promoting host biological control, bioremediation, growth, and development and may greatly facilitate the adaptation of hadal amphipods to extreme environments.

Metagenomic sequencing overcomes the hurdles of amplicon analysis because it analyzes total DNA extracted from samples and does not depend on target-specific primers. However, for the analysis of host-derived samples, this advantage of metagenomic sequencing is also vulnerable. Because the host genome is roughly a thousand times larger than an average bacterial genome, host DNA can quickly drown out microbial reads in samples containing even a relatively small number of host cells. In addition, the proportion of host cells to microbial cells varies widely by sampling site; for instance, fecal samples from healthy controls typically yield < 10% host genome-aligned reads, but the skin sample routinely contains >90% (Marotz et al., 2018). In the present study, the hadal amphipod mucosa samples were used for metagenomic sequencing. Because there was no available reference genome of hadal amphipods, we can only perform the host removal analysis using the reference genome from an available amphipod (*H. Azteca*) in combination with the strict parameters of BWA. Despite the efforts to align to a distant relative, it is likely that there is still host contamination present in the reads and which requires further improvements, such as improvements to sequencing methods (Marotz et al., 2018) and DNA extraction methods (Feehery et al., 2013; Fiedorová et al., 2019).

## Data Availability Statement

All sequencing data associated with this project were deposited in the National Center for Biotechnology Information (NCBI) Sequence Read Archive database (BioProject Accession Number: PRJNA648173; SRA Accession Number: SRR12315170-SRR12315178.

## Ethics Statement

Experimental protocols involved dead animals in this study.

## Author Contributions

QX conceived the experiments, led the whole project, and contributed to edits to the manuscript. JC analyzed the data and performed the biological experiments. BP designed the lander vehicle for sample collection. DG extracted the DNA. JC, DG, BP, and QZ collected the samples. JC and QX wrote the manuscript. All authors contributed to the article and approved the submitted version.

## Conflict of Interest

QZ was employed by the company Shanghai Rainbowfish Ocean Technology Co., Ltd. The remaining authors declare that the research was conducted in the absence of any commercial or financial relationships that could be construed as a potential conflict of interest.

## Footnotes

1 http://www.illumina.com

2 https://github.com/jstjohn/SeqPrep

3 https://github.com/najoshi/sickle

4 https://www.ncbi.nlm.nih.gov/genome/?term=hyalella+azteca

5 http://bio-bwa.sourceforge.net

6 https://github.com/voutcn/megahit

7 http://www.ncbi.nlm.nih.gov/Taxonomy/taxonomyhome.html/index.cgi?chapter=tgencodes#SG1

8 http://www.bioinformatics.org/cd-hit/

9 http://blast.ncbi.nlm.nih.gov/Blast.cgi

10 https://www.kegg.jp/

11 http://smart.embl-heidelberg.de/smart/set_mode.cgi?GENOMIC=1

## Supplementary Material

Supplementary Figure 1 Gene Ontology (GO)classification of the total non-redundant protein-coding genes from all intestinal samples.

Supplementary Figure 2 Extended error bar plot considering differences of GO functional categories between the three hadal amphipod species. Different colors indicate different groups. The rightmost is the *P* value.

Supplementary Figure 3 Phylogenetic and domain composition analysis of *MCPs* from different microbial species. The phylogenetic tree was analyzed by maximum likelihood with Tamura-Nei model. The bootstrap values were indicated on the branches of the tree. Protein domain prediction was performed by SMART tool. Different domain types are represented by different graphic shapes.

Supplementary Figure 4 Network plots showing correlations between probiotic effects and categories of predicted gene functions in hadal amphipods. Significant correlations between probiotic effects and functional gene categories are connected by nodes and straight lines. The size of the nodes and thickness of the lines represents the correlation strength. The larger the nodes and thicker the lines indicated the higher the correlations. The more lines indicate the closer correlations.

Supplementary Table 1 Summary of sequencing data for each sample.

Supplementary Table 2 Assembly result statistics of metagenomic datasets.

Supplementary Table 3 Expression profiles of the genes involved in the “Bacterial Chemotaxis” and “Flagellar Assembly” KEGG pathways.
