# Supplementary figures and images for: Metagenomic Insights Into the Structure and Function of Intestinal Microbiota of the Hadal Amphipods
Source: Front Microbiol. 2021 Jun 7;12:668989. doi: 10.3389/fmicb.2021.668989 (PMC8216301; doi:10.3389/fmicb.2021.668989)

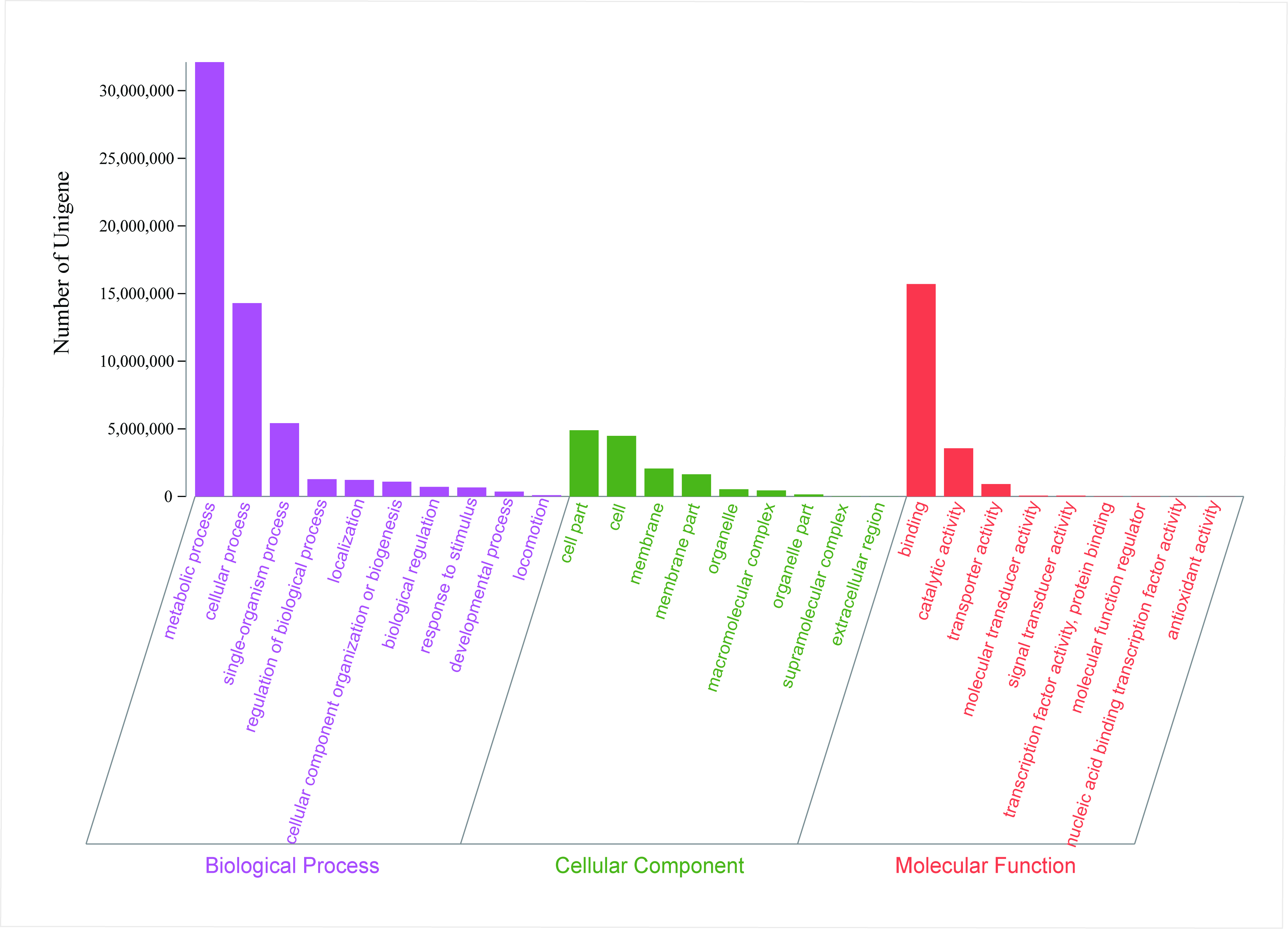

Supplement: Supplementary Figure 1 — Gene Ontology (GO)classification of the total non-redundant protein-coding genes from all intestinal samples. [file Image_1.JPEG]

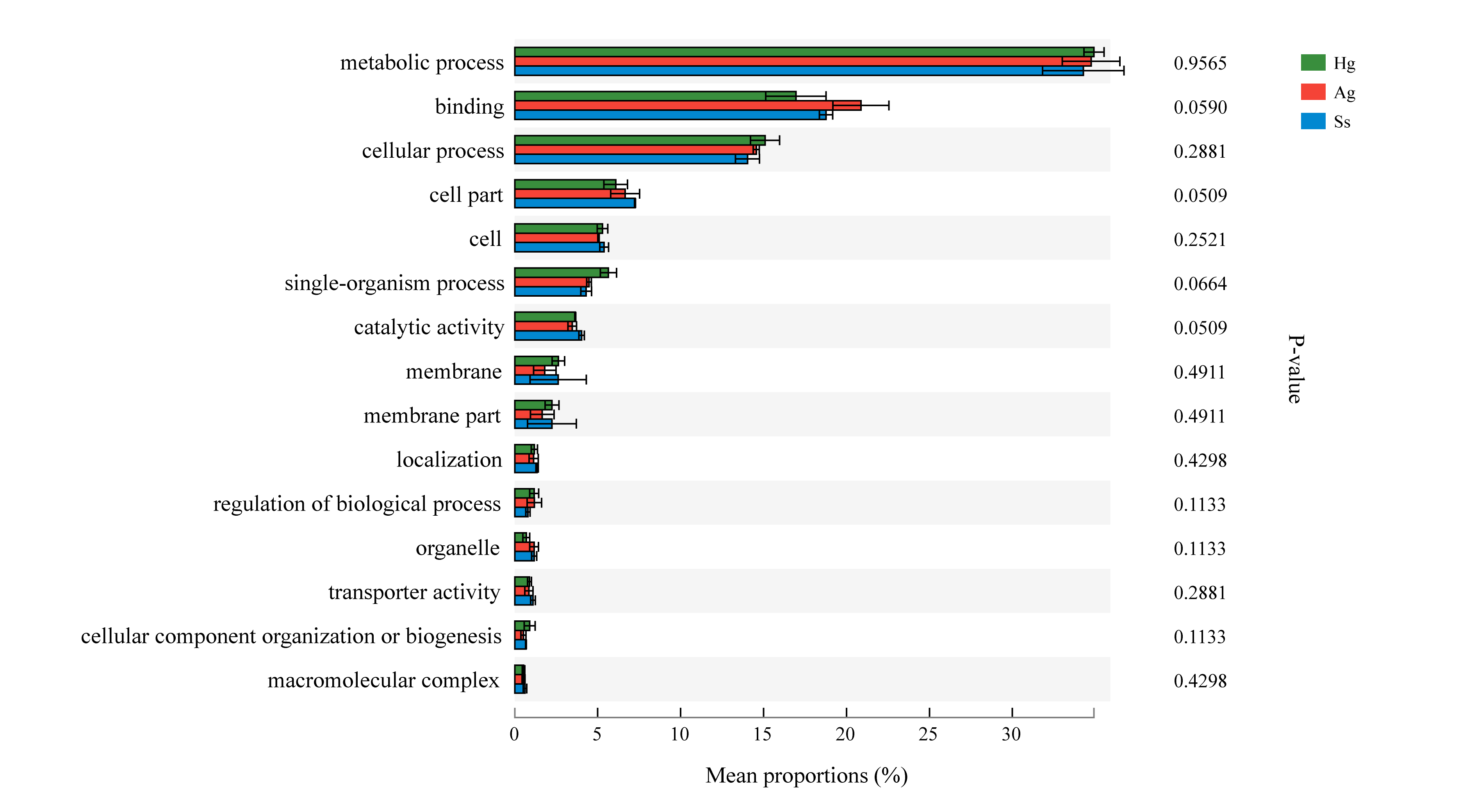

Supplement: Supplementary Figure 2 — Extended error bar plot considering differences of GO functional categories between the three hadal amphipod species. Different colors indicate different groups. The rightmost is the P value. [file Image_2.JPEG]

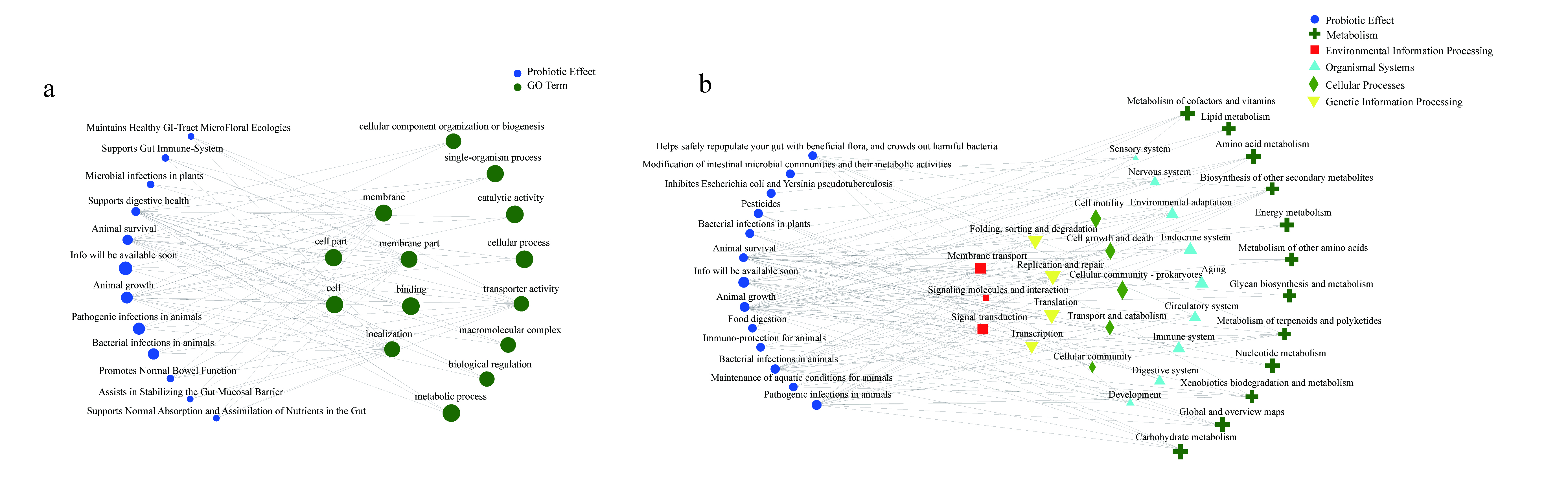

Supplement: Supplementary Figure 4 — Network plots showing correlations between probiotic effects and categories of predicted gene functions in hadal amphipods. Significant correlations between probiotic effects and functional gene categories are connected by nodes and straight lines. The size of the nodes and thickness of the lines represents the correlation strength. The larger the nodes and thicker the lines indicated the higher the correlations. The more lines indicate the closer correlations. [file Image_4.JPEG]
