# Supplementary material for: Metagenomic Insights Into the Structure and Function of Intestinal Microbiota of the Hadal Amphipods
Source: Front Microbiol. 2021 Jun 7;12:668989. doi: 10.3389/fmicb.2021.668989 (PMC8216301; doi:10.3389/fmicb.2021.668989)
Supplement: Supplementary Table 1 — Summary of sequencing data for each sample. [file Table_1.DOCX]

Supplementary Table S1. Summary of sequencing data for each sample.

| Samples | Raw reads | Clean reads | Percent in raw reads (%) | Optimized reads | Percent in raw reads (%) |
| --- | --- | --- | --- | --- | --- |
| Ag_1 | 49,966,314 | 47,220,908 | 94.51 | 46,131,064 | 92.32 |
| Ag_2 | 58,790,998 | 55,797,941 | 94.91 | 54,609,467 | 92.89 |
| Ag_3 | 54,453,540 | 51,683,129 | 94.91 | 50,461,338 | 92.67 |
| Hg_1 | 61,990,810 | 59,450,179 | 95.90 | 51,463,125 | 83.02 |
| Hg_2 | 55,475,450 | 52,788,456 | 95.16 | 44,861,234 | 80.87 |
| Hg_3 | 61,785,680 | 59,560,151 | 96.40 | 50,487,054 | 81.71 |
| Ss_1 | 59,414,250 | 55,727,840 | 93.80 | 53,163,483 | 89.48 |
| Ss_2 | 56,856,644 | 53,120,418 | 93.43 | 50,622,394 | 89.04 |
| Ss_3 | 71,790,672 | 68,528,116 | 95.46 | 66,234,160 | 92.26 |

Supplementary Table S2. Assembly result statistics of metagenomic datasets.

| Samples | Contig number | Assembly length (bp) | N50 (bp) | N90 (bp) | Max (bp) |
| --- | --- | --- | --- | --- | --- |
| Ag_1 | 434,883 | 218,494,372 | 503 | 329 | 23,068 |
| Ag_2 | 607,613 | 315,330,061 | 530 | 333 | 10,565 |
| Ag_3 | 529,040 | 272,576,661 | 522 | 332 | 62,950 |
| Hg_1 | 235,619 | 123,053,785 | 506 | 326 | 200,457 |
| Hg_2 | 248,320 | 124,308,885 | 486 | 325 | 25,400 |
| Hg_3 | 311,118 | 150,718,541 | 469 | 324 | 22,151 |
| Ss_1 | 256,891 | 117,865,562 | 447 | 322 | 16,664 |
| Ss_2 | 226,763 | 104,692,710 | 446 | 322 | 60,429 |
| Ss_3 | 876,945 | 409,948,392 | 467 | 327 | 57,268 |
