# Supplementary material for: Metagenomic Insights Into the Structure and Function of Intestinal Microbiota of the Hadal Amphipods
Source: Front Microbiol. 2021 Jun 7;12:668989. doi: 10.3389/fmicb.2021.668989 (PMC8216301; doi:10.3389/fmicb.2021.668989)
Supplement: Supplementary Table 2 — Assembly result statistics of metagenomic datasets. [file Table_2.DOCX]

Supplementary Table S2. Assembly result statistics of metagenomic datasets.

| Samples | Contig number | Assembly length (bp) | N50 (bp) | N90 (bp) | Max (bp) |
| --- | --- | --- | --- | --- | --- |
| Ag_1 | 434,883 | 218,494,372 | 503 | 329 | 23,068 |
| Ag_2 | 607,613 | 315,330,061 | 530 | 333 | 10,565 |
| Ag_3 | 529,040 | 272,576,661 | 522 | 332 | 62,950 |
| Hg_1 | 235,619 | 123,053,785 | 506 | 326 | 200,457 |
| Hg_2 | 248,320 | 124,308,885 | 486 | 325 | 25,400 |
| Hg_3 | 311,118 | 150,718,541 | 469 | 324 | 22,151 |
| Ss_1 | 256,891 | 117,865,562 | 447 | 322 | 16,664 |
| Ss_2 | 226,763 | 104,692,710 | 446 | 322 | 60,429 |
| Ss_3 | 876,945 | 409,948,392 | 467 | 327 | 57,268 |
