# Supplementary material for: Metagenomic Insights Into the Structure and Function of Intestinal Microbiota of the Hadal Amphipods
Source: Front Microbiol. 2021 Jun 7;12:668989. doi: 10.3389/fmicb.2021.668989 (PMC8216301; doi:10.3389/fmicb.2021.668989)
Supplement: Supplementary Table 3 — Expression profiles of the genes involved in the “Bacterial Chemotaxis” and “Flagellar Assembly” KEGG pathways. [file Table_3.DOCX]

Table S3 Expression profiles of the genes involved in the “Bacterial Chemotaxis” and “Flagellar Assembly” KEGG pathways

| KEGG pathway | Gene name | Hg_1 | Hg_2 | Hg_3 | Ss_1 | Ss_2 | Ss_3 | Ag_1 | Ag_2 | Ag_3 |
| --- | --- | --- | --- | --- | --- | --- | --- | --- | --- | --- |
| Bacterial Chemotaxis | methyl-accepting chemotaxis protein, MCP | 19180 | 17682 | 18324 | 9753 | 10524 | 8352 | 13029 | 8937 | 10262 |
|  | aerotaxis receptor, Aer | 2932 | 2783 | 2861 | 1473 | 1492 | 1376 | 1997 | 1362 | 1573 |
|  | chemotaxis protein methyltransferase, CheR | 2642 | 2356 | 2537 | 1327 | 1304 | 1385 | 1452 | 1265 | 1278 |
|  | chemotaxis family, response regulator, CheB | 4614 | 4374 | 4569 | 2790 | 2648 | 2494 | 2315 | 2278 | 2402 |
|  | chemotaxis family, sensor kinase, CheA | 4376 | 4438 | 4403 | 2303 | 2263 | 2404 | 2520 | 2195 | 2218 |
|  | purine-binding chemotaxis protein, CheW | 3782 | 3852 | 3721 | 1946 | 1913 | 2031 | 2130 | 1855 | 1874 |
|  | chemotaxis family, response regulator, CheV | 5342 | 5274 | 5429 | 3040 | 2790 | 2664 | 3307 | 2507 | 2835 |
|  | chemotaxis protein, CheZ | 2358 | 2478 | 2391 | 2251 | 2229 | 2305 | 1768 | 1492 | 1504 |
|  | chemotaxis protein, CheX | 930 | 1034 | 972 | 708 | 652 | 631 | 556 | 485 | 490 |
|  | chemotaxis family, response regulator, CheY | 3130 | 3201 | 3092 | 2617 | 2589 | 2688 | 1570 | 1742 | 1558 |
| Flagellar Assembly | flagellar biosynthetic protein, FliR | 2634 | 2594 | 2602 | 1361 | 1337 | 1420 | 1489 | 1297 | 1311 |
|  | flagellar motor switch protein, FliG | 2952 | 2919 | 2916 | 1725 | 1699 | 1492 | 1769 | 1554 | 1369 |
|  | flagellar assembly protein, FliH | 1956 | 1734 | 1832 | 1511 | 1993 | 1855 | 706 | 963 | 773 |
|  | flagellum-specific ATP synthase, FliI | 2986 | 2754 | 3050 | 2543 | 2516 | 3010 | 2688 | 2371 | 2486 |
|  | flagellar protein, FliZ | 794 | 732 | 697 | 310 | 403 | 278 | 449 | 391 | 395 |
|  | flagellin, Fli C | 7912 | 8021 | 7816 | 4188 | 4717 | 4367 | 4473 | 4197 | 3937 |
|  | flagellar hook-length control protein, FliK | 4532 | 4335 | 4577 | 2342 | 2391 | 2444 | 2562 | 2032 | 2255 |
|  | flagellar biosynthetic protein, FliP | 2276 | 2328 | 2248 | 1176 | 1156 | 1227 | 1287 | 1121 | 1133 |
|  | flagellar hook-associated protein 2, FliD | 6484 | 6631 | 6405 | 6350 | 6292 | 6497 | 5666 | 5194 | 5227 |
|  | flagellar hook-basal body complex protein, FliE | 940 | 961 | 929 | 486 | 477 | 507 | 531 | 463 | 468 |
|  | flagellar M-ring protein, FliF | 5238 | 5057 | 4674 | 3707 | 4660 | 4825 | 2961 | 2780 | 3007 |
|  | flagellar biosynthetic protein, FlhB | 2522 | 3079 | 2491 | 1003 | 1281 | 960 | 1126 | 942 | 1055 |
|  | chemotaxis protein, MotB | 5782 | 6013 | 5712 | 2388 | 2536 | 3118 | 3269 | 2848 | 2877 |
|  | chemotaxis protein, MotA | 4992 | 48c05 | 4931 | 2579 | 2535 | 2692 | 2822 | 2459 | 2484 |
|  | sodium-type polar flagellar protein, MotX | 2028 | 2174 | 1703 | 1048 | 1030 | 1094 | 1147 | 999 | 1009 |
|  | sodium-type flagellar protein, MotY | 2378 | 2332 | 2349 | 1529 | 1307 | 1282 | 1344 | 1571 | 1083 |
|  | flagellar biosynthesis protein, FlhA | 5844 | 5477 | 5573 | 3020 | 2967 | 3152 | 3304 | 2879 | 2908 |
|  | flagellar hook protein, FlgE | 4416 | 4416 | 4362 | 2382 | 2142 | 2421 | 2097 | 1775 | 1998 |
|  | flagellar basal-body rod protein, FlgG | 2358 | 2411 | 2329 | 2218 | 2397 | 1972 | 2333 | 2161 | 2173 |
|  | flagellar L-ring protein precursor, FlgH | 2410 | 2065 | 2381 | 1045 | 1324 | 1323 | 1563 | 1387 | 1199 |
|  | flagellar basal-body rod protein, FlgC | 1416 | 1548 | 1499 | 764 | 819 | 704 | 1001 | 997 | 705 |
|  | flagellar hook-associated protein, 1 FlgK | 4468 | 3969 | 4514 | 2309 | 2369 | 2810 | 2563 | 1901 | 2223 |
|  | flagellar P-ring protein precursor, FlgI | 4008 | 4299 | 3959 | 3071 | 3035 | 3161 | 1266 | 974 | 994 |
|  | flagellar basal-body rod modification protein, FlgD | 2306 | 2258 | 2278 | 2192 | 2171 | 2044 | 1704 | 1536 | 1448 |
|  | flagellar hook-associated protein 3, FlgL | 4824 | 4933 | 4765 | 3493 | 3449 | 3602 | 2927 | 2376 | 2601 |
|  | negative regulator of flagellin synthesis, FlgM | 772 | 790 | 763 | 1399 | 1392 | 1416 | 436 | 380 | 384 |
|  | flagellar basal-body rod protein, FlgB | 1368 | 1399 | 1351 | 1707 | 1695 | 1738 | 1773 | 1674 | 1681 |
|  | flagella synthesis protein, FlgN | 1536 | 1371 | 1517 | 1794 | 1780 | 1828 | 868 | 757 | 764 |
|  | flagellar basal-body rod protein, FlgF | 2022 | 1868 | 1997 | 1045 | 1027 | 1090 | 1143 | 996 | 1006 |
